# Metabolomic analysis with GC-MS to reveal potential metabolites and biological pathways involved in Pb & Cd stress response of radish roots

**DOI:** 10.1038/srep18296

**Published:** 2015-12-17

**Authors:** Yan Wang, Liang Xu, Hong Shen, Juanjuan Wang, Wei Liu, Xianwen Zhu, Ronghua Wang, Xiaochuan Sun, Liwang Liu

**Affiliations:** 1 National Key Laboratory of Crop Genetics and Germplasm Enhancement; Engineering Research Center of Horticultural Crop Germplasm Enhancement and Utilization, Ministry of Education of P.R. China; College of Horticulture, Nanjing Agricultural University, Nanjing 210095, P.R. China; 2 The National Agro-Tech Extension and Service Center, Beijing 100125, P.R. China; 3 Department of Plant Sciences, North Dakota State University, Fargo, ND 58108, USA

## Abstract

The radish (*Raphanus sativus* L.) is an important root vegetable crop. In this study, the metabolite profiling analysis of radish roots exposed to lead (Pb) and cadmium (Cd) stresses has been performed using gas chromatography-mass spectrometry (GC-MS). The score plots of principal component analysis (PCA) and partial least squares-discriminate analysis (PLS-DA) showed clear discrimination between control and Pb- or Cd-treated samples. The metabolic profiling indicated Pb or Cd stress could cause large metabolite alteration mainly on sugars, amino acids and organic acids. Furthermore, an integrated analysis of the effects of Pb or Cd stress was performed on the levels of metabolites and gene transcripts from our previous transcriptome work in radish roots. Kyoto Encyclopedia of Genes and Genomes (KEGG) pathway analysis of integration data demonstrated that exposure of radish to Pb stress resulted in profound biochemical changes including carbohydrate metabolism, energy metabolism and glutathione metabolism, while the treatment of Cd stress caused significant variations in energy production, amino acid metabolism and oxidative phosphorylation-related pathways. These results would facilitate further dissection of the mechanisms of heavy metal (HM) accumulation/tolerance in plants and the effective management of HM contamination in vegetable crops by genetic manipulation.

Heavy metal (HM)-pollution represents a major environmental hazard to human health due to the accelerating industrial developments in recent decades^1^,^2^. Among the non-nutrient heavy metals, lead (Pb) and cadmium (Cd) are the most common and widespread contaminants, which can be absorbed by plants through contaminated soil and water and then enter into the food chain^3^,^4^. In plants, heavy metal toxicity such as Pb and Cd could induce a number of morphological, physiological and biochemical defects including transpiration and photosynthesis alteration, carbohydrate metabolism imbalance and production of secondary stresses like nutrition stress, water deficit and oxidative stress^4^,^5^,^6^. Nevertheless, plants also have evolved diverse endogenous mechanisms to cope with the toxic effects of heavy metals including generating signal sensing and transduction proteins, activating transport systems and biosynthesis of chelating compounds^7^,^8^,^9^. However, systematically understanding the heavy metal toxicity mechanism and identifying the cellular or biochemical targets underlying plant physiological responses are complex and challenging.

Recently, the arrival of ‘omics’ approaches have been widely used in modern biology aimed at massively characterising the molecular mechanisms of living systems at different levels, which can give a system-wide view of understanding the various layers of the cellular architecture underlying function of biological networks^10^,^11^,^12^. Metabolomics is one of the last additions to the ‘omics’ wave, which can provide an approach to unravel the complex mechanisms by measuring many metabolites participating in various biochemical processes and across many biological systems^13^,^14^. There are various analytical platforms including gas chromatography-mass spectrometry (GC-MS), liquid chromatography (LC)-MS, capillary electrophoresis (CE)-MS and nuclear magnetic resonance spectroscopy (NMR) commonly used in plant metabolomics research^15^,^16^. Increasing evidence has revealed that metabolomics studies are playing important roles in the post-genomic area for characterising physiological responses to various metal stresses in plants^17^,^18^,^19^,^20^,^21^. Metabolite profiling was analyzed in response to sulfur nutritional stress using GC-MS and LC-MS in *Arabidopsis thaliana*^15^. Metabolic consequences of cesium (Cs) stress were also reported in *Arabidopsis* based on NMR-based metabolomics^18^. Metabolic changes were also investigated in *Silene cucubalus*^19^, *Solanum lycopersicum*^20^ and *Populus tremula*^21^ upon Cd stress.

Radish (*Raphanus sativus* L.), belonging to the family *Brassicaceae*, is an economically important vegetable crop with an edible taproot. Because the root is considered the vulnerable part that is affected by some HMs, such as Cd and Pb^22^,^23^, it has become of vital importance to investigate the HM-response mechanisms and explore the molecular regulatory network of tolerance and homeostasis in radishes. From our previous studies, two cDNA libraries were construed from Pb-stressed and Pb-free roots of radish seedlings using Solexa sequencing technology. A set of differentially expressed genes, which were predominately involved in defense responses in cell walls, glutathione metabolism-related processes, and carbohydrate metabolism-related pathways were detected^24^. Twenty-four Cd-responsive miRNAs were identified, and several miRNAs have been found to target transcripts associated with metal transport, signaling and sequestration in radish roots when exposed to Cd stress^25^. Nevertheless, there has been no report on investigating the metabolite changes in response to HM stress by employing metabolomics technology in radish.

GC-MS is the most widely used technique for plant metabolomics research to date, especially for facilitating the identification and quantification of the metabolites involved in the central pathways of primary metabolism such as sugars, sugar alcohols, amino acids, organic acids and polyamines^26^. In this study, the metabolite profiling analysis in the roots of radish after the short-term exposure to Pb or Cd (72 h) stress has been performed using GC-MS analysis. Multivariate statistical tools including unsupervised principle component analysis (PCA) and supervised partial least squares-discriminate analysis (PLS-DA) have been employed to reduce and visualize the complex metabolomics datasets. From that, a broad range of Pb- and Cd-responsive metabolites was quantified, and the changes in several metabolic pathways were highlighted. Additionally, to reveal the integrative biochemical networks of the radish in response to Pb or Cd stress, the metabolomic data were integrated with our previous transcriptomic data, which provided a more global view of the molecular and cellular changes elicited by Pb and Cd exposure in radish. This work comprehensively provides the framework for a better understanding of the mechanisms that govern plant cell response to HM-induced stress in radish at a molecular level, and the resulting information would facilitate the effective management of HM contamination in vegetable crops by genetic manipulation.

## Results

### Metabolic changes in response to Pb and Cd stresses in radish

**Figure 1:**
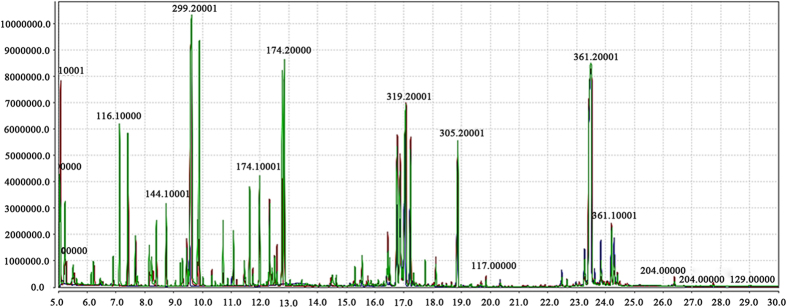
The overlay total ion chromatograms (TICs) of the three groups by gas chromatography-mass spectrometry (GC-MS) analysis. The red, blue and green peaks respectively indicated a representative sample from Pb-treated, Cd-treated and control groups of radish roots.

In our preliminary experiment studies, different concentrations of Pb(NO_3_)_2_ (100, 200, 400, 800, 1,000 and 1,500 mg∙L^−1^) and CdCl_2_∙2.5H_2_O (50, 100, 150, 200, 400 and 500 mg · L^−1^) were set to investigate the changes of visible physiology symptoms in an advanced inbred radish line ‘NAU-RG’ with different temporal durations. Interestingly, no obvious morphologic differences were found among individuals when exposed to low dose of heavy metal treatment (0 to 800 mg · L^−1^ Pb(NO_3_)_2_ and 0 to 200 mg · L^−1^ CdCl_2_∙2.5H_2_O) for a maximum of 72 h, while the plants were seriously hampered and grew abnormally when exposed to 1,500 mg · L^−1^ Pb(NO_3_)_2_ and 500 mg · L^−1^ CdCl_2_∙2.5H_2_O. Therefore, we selected the concentrations of Pb(NO_3_)_2_ at 1,000 mg · L^−1^ (Pb1000) and CdCl_2_∙2.5H_2_O at 400 mg · L^−1^(Cd400) for the metabolomic analysis. Additionally, a control group was defined using non-treated seedlings (CK). Metabolites were extracted from taproot samples in eight replicate pools of plants for each of the three experimental groups including Pb1000, Cd400 and CK and were analyzed by GC-MS. The total ion chromatograms (TICs) of all samples (except one sample from Pb1000 group) demonstrated a strong signal, large peak capacity, and reproducible retention time, indicating the reliability of metabolomic analysis. Obvious chromatographic differences were observed between different sample groups, and a total of 1,104 types of metabolites were identified. Typical TICs from each group were shown in Fig. 1 and Supplementary Fig. S1.

**Figure 2:**
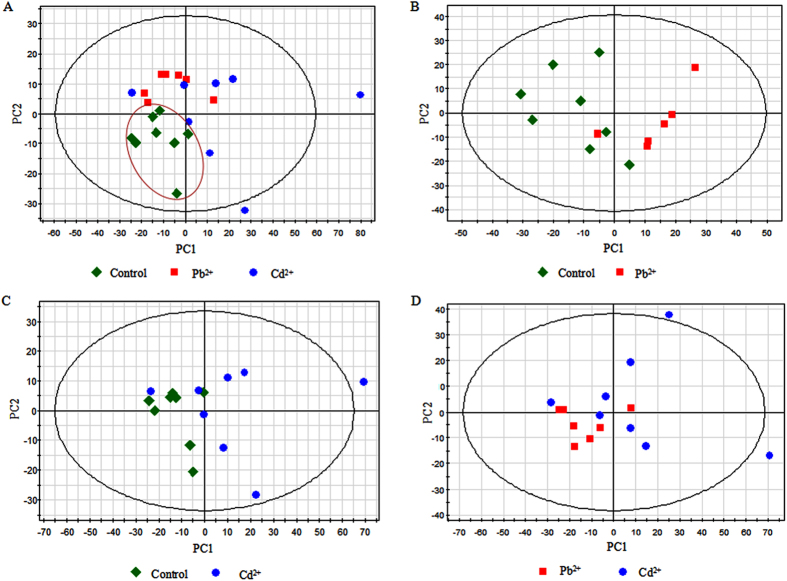
Principal component analysis (PCA) score plots of metabolic profiles in radish roots under the exposure of Pb and Cd stress. (**A**) PCA score plot for control (green), Pb-treated (red) and Cd-treated (blue) samples, (**B**) PCA score plot for control (green) and Pb-treated (red) samples, (**C**) PCA score plot for control (green) and Cd-treated (blue)samples, (**D**) PCA score plot for Pb-treated (red) and Cd-treated (blue) samples.

In order to reduce the dimensionality of the data and visualize samples grouping, an unsupervised multivariate data analysis method PCA was performed on the GC-MS data generated from Pb1000, Cd400 and CK groups. A PCA model was created with four principal components (PCs), which had explanation and predictability values of 74.8% and 51.9%, respectively (Table 1). The score plot of the first two PCs was shown in Fig. 2A. Most of the data were lying inside the 95% confidence region (Hotelling T^2^ ellipse). The results of the PCA for the three group samples indicated that an obvious separation between the control (CK) and treated samples (Pb1000 and Cd400) was detected, while no clear difference was observed between the Pb- (Pb1000) and Cd-treated (Cd400) groups (Fig. 2A). In order to confirm this tendency, every two of these three groups were analyzed by PCA with similar results obtained. According to the PCA models, two, three and two PCs were gained from the control and Pb-treated, control and Cd-treated and Pb- and Cd-treated samples comparisons, respectively. The R^2^X and Q^2^ values (goodness of prediction) are shown in Table 1, indicating that all the models could predictably explain the differences between groups. However, no distinct boundary between every two groups could be identified in the PCA score plots (Fig. 2B–D).

**Figure 3:**
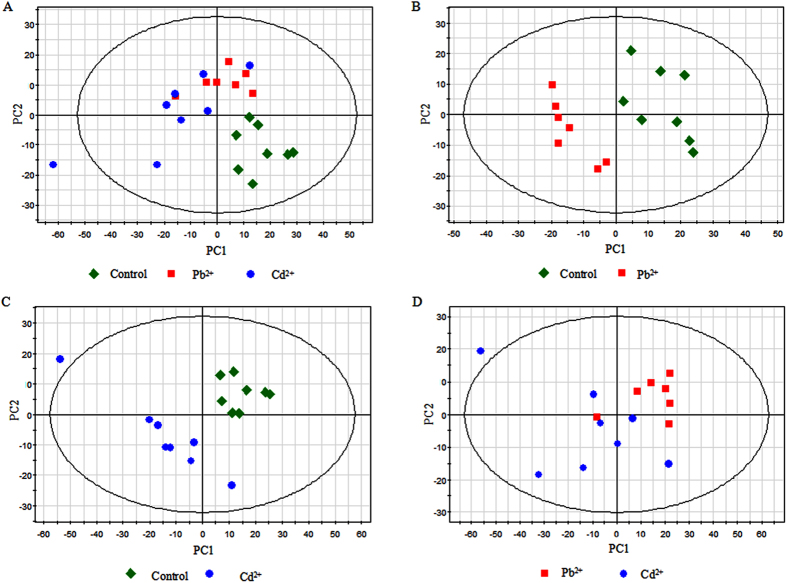
Partial least squares-discriminate analysis (PLS-DA) score plots of metabolic profiles in radish roots under Pb and Cd exposure. (**A**) PLS-DA score plot for control (green), Pb-treated (red) and Cd-treated (blue) samples, (**B**) PLS-DA score plot for control and Pb-treated (red) samples, (**C**) PLS-DA score plot for control (green) and Cd-treated (blue) samples, (**D**) PLS-DA score plot for Pb- treated (red) and Cd-treated (blue) samples.

Compared with PCA, PLS-DA is a supervised method, which could classify the observations into the group from giving the largest predicted indicator variable. From that, two PCs (R^2^X = 0.560, R^2^Y = 0.516, Q^2^ = 0.343) were indicated among the three groups, and the classification among these three groups was improved in the score plot (Fig. 3A). More satisfactory modeling and prediction results with two PCs were obtained when data were analyzed only using the control and the Pb- or Cd-treated samples (R^2^Y > 0.9, Q^2^ > 0.6) (Table 1), and both Pb- and Cd-treated taproot samples were clearly separated from the control along the PC1 (Fig. 3B,C). As shown in Fig. 3D, Pb- and Cd-treated samples could be separated in the PLS-DA score plot with two PCs, despite minor overlap. Nevertheless, the R^2^Y and Q^2^ values were only 0.476 and 0.092, respectively (Table 1), indicating a less metabolic change between these two treated samples when compared with the control.

### Identification of the Pb- or Cd-responsive metabolites in radish roots

The altered metabolites were found from the line plots of the X-loadings of the first component of the PLS-DA pairwise comparison models. It was reported that the variable importance in the projection (VIP) values greater than 1 were considered the most relevant metabolites for explaining the responses^27^. On the basis of the parameter VIP > 1, 29 Pb- and 34 Cd-responsive metabolites with significant changes (student’s T-test *P* < 0.05), were respectively identified (Supplementary Table S1 and Supplementary Table S2). Among them, three compounds were unknown, and one compound was a type of steroid that could not be accurately identified. The changed metabolites were mainly sugars, amino acids, and organic acids (Tables 2 and 3). Compared to the non-treated control, 10 metabolites increased and 18 decreased in levels of the radish roots after Pb exposure, while only one increased and 32 metabolites decreased by Cd treatment.

**Figure 4:**
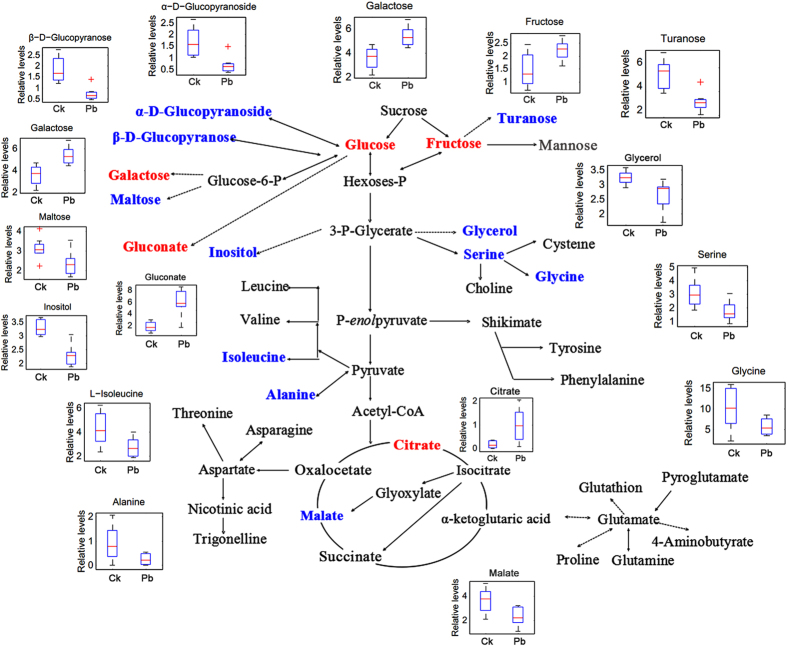
Metabolic changes involved in the primary pathways of radish roots under Pb exposure. The significantly up- and down-regulated (*P* < 0.05) metabolites were indicated in red and blue, respectively.

**Figure 5:**
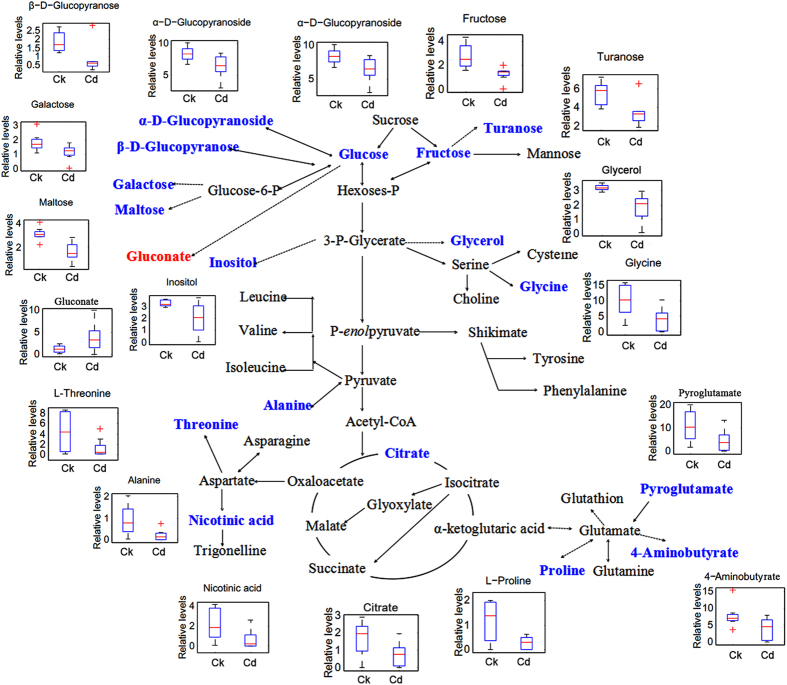
Metabolic changes involved in the primary pathways of radish roots under Cd exposure. The significantly up- and down-regulated (*P* < 0.05) metabolites were indicated in red and blue, respectively.

Consistent with the results of PLS-DA score plots, a different but overlapping metabolite-response trend to Pb and Cd stress was determined in radish. Furthermore, the altered metabolites involved in the primary metabolism of glycolysis and citric acid cycle and the linking metabolites to amino acid synthesis in radish roots under Pb or Cd stress were respectively summarized on a simplified metabolic map (Figs 4 and 5). As shown in Figs 4 and 5, the accumulation of gluconate was both enhanced under the two treatments, while coordinated decreases in the content of five sugars (maltose, inositol, turanose, α-D-glucopyranoside and β-D-glucopyranose), two amino acids (alanine and glycine), three organic acids (acetimidic acid, decanedioic acid and oxalic acid), glycerol, hydroxylamine, phosphoric acid and sitosterol were identified. However, the contents of three sugars (fructose, galactose and glucose), three organic acids (citrate, hexadecanoic acid and octadecanoic acid), and a type of steride were increased in response to Pb treatment but decreased to Cd treatment. Furthermore, induced accumulation of α-linolenic acid and declined contents derived from malate, serine and isoleucine were identified by Pb treatment, while no significant difference in the contents of these metabolites was detected after exposure of Cd. In contrast, the levels of α-D-glucopyranoside, proline, threonine, pyroglutamate, 4-aminobutyrate, pyrophosphate, nicotinic acid, cholesterol, monostearin and two unknown compounds were decreased by Cd rather than Pb exposure.

### Pathway mapping and the metabolite-to-metabolite network visualization

All of the changed metabolites affected by Pb and Cd stresses were mapped to the biological pathways involved in the KEGG database, which were assigned to 49 pathways in either treatment (Supplementary Tables S3 and S4). The most statistically enriched pathways were analyzed with a Bonferroni correction (*P* ≤ 0.05). The results showed that nine and four pathways were enriched with changed metabolites, as a result of Pb and Cd exposure, respectively (Table 4). Among them, three, including galactose metabolism, starch and sucrose metabolism, and aminoacyl-tRNA biosynthesis, were enriched for both the Pb- and Cd-regulated metabolites. Furthermore, using all the altered metabolites as inputs, we constructed the metabolite-to-metabolite interaction networks comprising 14 and 16 metabolites for the Pb- and Cd-stress exposure in radish roots, respectively (Supplementary Fig. S2). Both the networks could be divided into two sub-clusters. One sub-cluster consisted of compounds mainly involved in carbohydrate and glycan biosynthesis and metabolism, and the other was composed of several kinds of acidic compounds including amino acids, organic acids, and inorganic acids.

### Integrated transcriptome and metabolome analysis of radish in response to Pb or Cd stress

**Figure 6:**
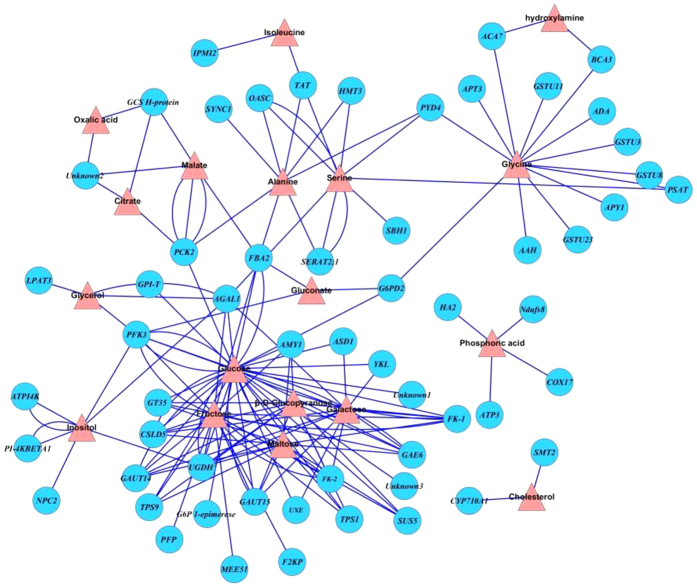
The gene-to-metabolite networks involved in radish root response to Pb stress. The annotations for the related Pb-responsive genes were listed in Table S6.

**Figure 7:**
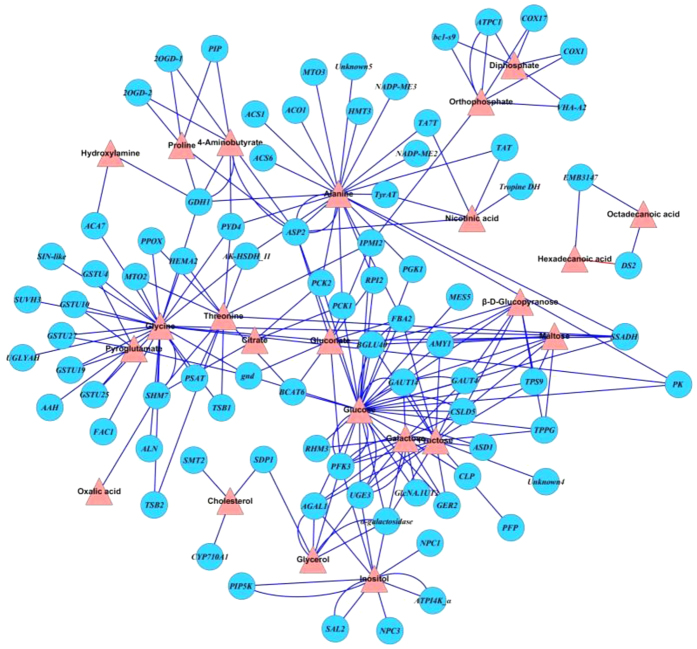
The gene-to-metabolite networks involved in radish root response to Cd stress. The annotations for the related Cd-responsive genes were listed in Table S8.

To gain a deeper understanding of the molecular mechanism of heavy metal tolerance and homeostasis in radish, an integrated analysis of the effects of Pb or Cd stress was performed on the levels of metabolites and gene transcripts from our previous transcriptome work in radish roots^24^,^25^. All the Pb- or Cd-responsive genes and metabolites were separated, and a gene-to-metabolite network was constructed each for the Pb-(Fig. 6) and Cd-stress exposure (Fig. 7). During Pb exposure, most of intersected pathways between differentially expressed genes and altered metabolites were involved in carbohydrate metabolism (i.e., starch and sucrose metabolism, fructose and mannose metabolism and the pentose phosphate pathway), energy metabolism (i.e., citrate cycle and glycolysis/gluconeogenesis), and glutathione metabolism (Supplementary Table S5). The gene-to-metabolite network consisted of 18 metabolites and 58 genes (Fig. 6 and Supplementary Table S6). As shown in Fig. 6, the expression of several genes encoding enzymes including glutathione S-transferase (*GSTU3*, *GSTU8*, *GSTU11* and *GSTU23*), carbonate dehydratase (*ACA7* and *BCA3*), amidohydrolase (*AAH*), adenine phosphoribosyl transferase (*APT3*), glucose-6-phosphate dehydrogenase (*G6PD2*), phosphoserine aminotransferase (*PSAT*), adenosine deaminase (*ADA*), and nucleoside triphosphatase (*APY1*) were correlated with the regulation of glycine content. Isopropylmalate isomerase- and tyrosine aminotransferase-encoding genes (*IPMI2* and *TyrAT*) were found to be responsible for the decrease of isoleucine after the treatment of Pb. Six genes simultaneously participated in the regulation of alanine and serine, while three aminotransferase (*HMT3*, *TyrAT* and *SERAT2;1*), one cysteine synthase (*OASC*), one pyrimidine (*PYD4*), and one fructose-bisphosphate aldolase (*FBA2*) gene showed involvement. Additionally, most other genes including those encoding amylase (*AMY1*), 1,4-beta-D-xylan synthase (*CSLD5*), galacturonosyltransferase (*GAUT14* and *GAUT15*), trehalose-phosphate synthase (*TPS1* and *TPS9*), UDP-D-glucuronate 4-epimerase (*GAE6*), UDP-glucose 6-dehydrogenase (*UDG4*), galactosidase (*AGAL1*), fructose-bisphosphate aldolase (*FBA2*), phosphoenolpyruvate carboxykinase (*PCK2*), sucrose synthase (*SUS5*), glucan phosphorylase (*PHS1*), fructose-2,6-bisphosphatase (*F2KP*) and phosphofructokinase (*PFK*), *Chitinase-like* and *G6PD2* were involved in the regulation of the concentration of storage carbohydrate (such as fructose, glucose, maltose and glucopyranose).

All of the intersected pathways in response to Pb exposure were included in those pathways in response to Cd exposure. Also, some specific metabolites and genes responding to Cd stress were involved in pathways participating in oxidative phosphorylation, amino acid-related metabolism, and nitrogen metabolism (Supplementary Table S7). The corresponding gene-to-metabolite network under Cd exposure was mapped in Fig. 7, which included 23 metabolites and 82 genes (Supplementary Table S8). Similar genes were found to regulate the accumulation of inositol (i.e., *NPC*s, *SAL2*, *PFK3* and *ATP14K-α*) and cholesterol (i.e., *CYP707A1* and *SMT2*) in response to both Pb and Cd stress. Five *GSTU* genes, *SHM7* (serine hydroxymethyltransferase-encoding gene), and *6PGD* (6-phosphogluconate dehydrogenase-encoding gene) were reported to affect the accumulation of both glycine and pyroglutamate. Genes *PYD4*, *PPOX*, *HEMA2*, *MTO2*, *SHM7*, *PSAT*, *TSB* and *AK-HSDH* were responsible for the regulation of glycine and threonine. In addition, *AAH*, *ACA7*, *SIN-like*, *SUVH3*, *UGLYAH*, *FAC1* and *GDH1* were involved in glycine metabolism. Among multiple genes involved in the Cd-altered alanine metabolism, there were five genes encoding aminotransferases and five genes involved in the ethylene synthesis pathway. Similar to the situation in the Pb-related gene-to-metabolite network (Fig. 6), complex relationships were also observed in the Cd-regulated storage carbohydrate metabolism. In addition to *PFK3*, *AGAL1*, *ASD1*, *TSP9*, *CSLD5*, *GAUT14*, *AMY1*, *FBA2*, *chitinase-like*, *PCK2* and *MEE51*, which also respond to Pb, genes encoding 6-dehydratase (*RHM3*), UDP-glucose (*UDG4* and *UGE3*), GDP-L-fucose synthase (*GER2*), trehalose-6-phosphate phosphatase (*TPPG*), glucosidase (*BGLU40*), and ribose 5-phosphate isomerase (*RPI2*) were also responsible for Cd-regulated carbohydrate metabolism.

## Discussion

Radish, an important vegetable crop, can uptake and accumulate heavy metals (HMs) including Pb and Cd in the root^28^,^29^. Many previous studies have reported the defense and damage of radish response to HMs at morphological, physiological and transcriptional levels^30^,^31^,^32^,^33^. However, there are no reports to investigate the metabolite changes of radish under the HMs exposure using metabolomics technology, which has been regarded as an important research field in the post-genomic area, especially for plant physiological responses to various stresses^34^,^35^,^36^. In this study, we report a comprehensive analysis of metabolic changes in radish responding to heavy metal (Pb or Cd) stress using a GC-MS-based metabolomics approach. The results indicated that heavy metal stress could cause many metabolite alterations, mainly alterations of sugars, amino acids, and organic acids. A KEGG pathway analysis integrating transcriptomic and metabolomic data demonstrated that exposure of radish to Pb stress resulted in profound biochemical changes including carbohydrate metabolism, energy metabolism and glutathione metabolism, while the treatment with Cd caused significant variation in energy production, amino acid metabolism and oxidative phosphorylation-related pathways. This study is the first report on the systematic identification and characterization of metabolic changes in response to HM stress in radish using metabolomic analysis.

### Metabolic responses of radish to Pb and Cd stresses

Physiological processes such as photosynthesis and respiration have been shown to be very sensitive to heavy metals in higher plants^37^. In our previous studies, chlorosis and growth inhibition has been observed as a primary toxicity symptom for radish under the high levels of heavy metals exposure such as Pb and Cd^30^,^33^, which may be attribute to the consequence of inhibited synthesis of chlorophyll, increased chlorophyll degradation and altered the levels of carbohydrate in the presence of metals. However, plants also have evolved intricate mechanisms to respond and defend the HM toxicity. For example, it was reported that the photoassimilates were stored in the form of hexoses and complex sugars under the HM stress, which can act as the osmoprotectants playing critical roles in osmotic adjustments or protection of cell constituents^21^. In the present study, the accumulations of glucose, galactose and fructose were enhanced under Pb treatment, while the contents of maltose, turanose, α-D-glucopyranoside and β-D-glucopyranose were reduced in the radish roots, suggesting that photoassimilates were stored as hexoses in radish after exposure to Pb. As shown in Fig. 4, the carbon flux mainly concentrated in the high diffusion rate of sugars (the upregulation of galactose, glucose and fructose) and glycolysis. The results may be attributed that the Pb stress has a negative effect on respiration^38^, therefore the radish roots need to strengthen the glycolysis to cope with the stress.

Chelation of toxic heavy metal ions by low molecular weight organic acids (LMWOAs) is another commonly effective mechanism for the HM defense system^39^. Many studies have demonstrated that citrate, malate and oxalate could play central roles in case of tolerance to aluminum (Al) stress^40^,^41^,^42^,^43^. Additionally, citrate, oxalate and gluconate were observed to be significantly proficient under the Cd exposure^44^, and a positive correlation was also established between Pb and citrate concentrations in xylem sap of *Sesuvium portulacastrum* and *Brassica juncea*^45^. In this study, the altered organic acids involved in the primary metabolism of radish roots under Pb or Cd stress (Figs 4 and 5) were those including gluconate, citrate and malate. The content of gluconate was both enhanced under the two treatments, while up-regulated citrate and down-regulated malate were only shown during the Pb stress. In addition, some other organic acids which were not involved in the primary metabolism were significantly accumulated such as hexadecanoic acid, linoleic acid, α-linolenic acid and octadecanoic acid. These results indicated that LMWOAs were associated with HMs stress in case of radish. Particularly under the stimulus of Cd stress, carbon flow mainly concentrated in gluconate which revealed that the radish roots may be more inclined to use gluconate to alleviate the Cd toxicity (Fig. 5).

Many plants have been shown to accumulate proline and other amino acids when exposed to heavy metals, which could affect synthesis and activity of the antioxidant enzymes, regulation of ion transport, modulating stomatal opening and sequestering toxic cadmium ions in the cytosol or vacuole^21^,^46^. However, in our study, the content of all the altered amino acids were both decreased and the carbon fluxes were less flow to the process of amino acid synthesis upon the Pb (Fig. 4) or Cd exposure (Fig. 5) in radish. The result revealed that high level of Pb or Cd inhibited the synthesis of amino acids, which may be associated with the reduced activity of antioxidant enzymes (such as SOD, POD, CAT and APX) under the high concentrations of metal ions exposure reported from our previous studies^30^,^33^.

### Gene-to-metabolite networks of radish in response to Pb and Cd stresses

Integration of transcriptomics and metabolomics data can better elucidate the gene functions and metabolic pathways in biology^47^. In the current study, the gene-to-metabolite networks of radish responding to Pb and Cd stress were respectively constructed, which exhibited a complex regulation mechanism such as one compound was affected by several genes and one gene regulated many metabolites. In addition, the primary metabolic and transcript changes were summarized on a simplified metabolic map (Supplementary Figs S3 and S4) according to the results of overlapping pathways involved in the KEGG metabolic database, which could strongly exhibit the direct metabolic relationship upon Pb and Cd exposure in radish roots. As shown in Supplementary Fig. S3 and S4, the expression of genes involved in glycolysis such as *PFK3*, *FBA2* and *PGK1* were affected by both the treatments of Pb and Cd stress. However, the contents of their direct products β-D-fructose-1, 6 P2, glyceraldehyde-3P and glycerate-3P were not influenced, suggesting that a complex regulation mechanism may exist.

Many studies reported that the glutathione (GSH) metabolism could play critical roles in heavy metal stress tolerance^48^,^49^. The *GSTU* gene families were the plant-specific tau class encoding glutathione S-transferases (GST), which were verified to participate in the conversion from glutathione and glycine^50^. As shown in Supplementary Figs S3 and S4, different members in the *GSTU* gene family were found to be differentially expressed and the content of glycine were both decreased under the Pb or Cd exposure of radish. Due to the diversity of structural classes of metabolites in plants, there is so far no single technique to identify and quantify all of them^15^. Here, the most critical biological thiols compound involved in GSH metabolism such as GSH, phytochelatins (PCs) and other sulfides could not be successfully detected by the GC-MS. In the previous studies, all the quantification of GSH and PCs were carried out through a targeted-metabolic-profiling analysis by LC-MS method^48^,^51^. In order to detect a wide range of metabolites, other techniques including LC-MS would be employed to investigate the metabolic profiles in radish upon heavy metal stress, and then more comprehensive profiles would be obtained with the different techniques.

In summary, HM-induced metabolomic responses by GC-MS analysis for radish roots during Pb and Cd stress showed different but overlapping metabolic alterations mainly on sugars, amino acids and organic acids. By integrated analysis of transcript and metabolic profiles, it demonstrate that exposure of radish to Pb or Cd stress resulted in profound biochemical pathway changes, and the integrative gene-to-metabolite networks were revealed in this study. These findings may provide useful information for understanding the molecular mechanisms involved in radish responding to HM-induced stresses, which would further facilitate the effective management of HM contamination in vegetable crops by genetic manipulation.

## Methods

### Plant materials

Seeds of an inbred line of radish (*Raphanus sativus* L.), ‘NAU-RG’ were surface-sterilized for 2 min with 2.5% NaClO followed by washing and soaking in sterile distilled water. After germinating in the dark at 25 °C, the seeds were transferred into culture bowls in a growth chamber (24/16 °C day/night temperature, 14/10 h light/dark). Seedlings with four fully expanded true leaves were transplanted into the culture pots, which were filled with modified half-strength Hoagland nutrient solution, as previously described^24^. After two weeks, the seedlings were treated with either 0, 1,000 mg · L^−1^ Pb(NO_3_)_2_, or 400 mg · L^−1^ CdCl_2_∙2.5H_2_O for 72 h. Eight biological replicates from each treatment were sampled and handled according to the reported descriptions^24^,^25^,^52^. For each replicate with five seedlings, nearly equal amount of samples from three randomly selected individual plants were pooled. All the harvested radish taproot samples were immediately frozen in liquid nitrogen and then stored at −80 °C for two weeks before metabolite extraction.

### Metabolite extraction from the radish taproots

Metabolites were extracted according to previous studies with some modifications^53^,^54^. Taproots were immediately plunged into liquid nitrogen to store for further analysis. Briefly, about 50 mg of each frozen samples were transferred into a 2-mL centrifuge tube, and then, 1mL of 100%-methanol (pre-cooled at −20 °C) was added to each tube. The mixtures were vortexed and ground by a 70-Hz grinding mill system (Jinxin Biotech Ltd., Shanghai, China) for 5 min. The homogenate was treated withultra-sonication for 30 min at 70 °C. Subsequently, the tubes were centrifuged at 14,000 g at 4 °C for 10 min. Of the supernatant, 0.4 mL was decanted into a 2-mL screw-top tube, including 10 μL internal standards (0.02 mg∙mL^−1^ 3,4-dichloropheny- lalanine in methanol), 200 μL chloroform (pre-cooled at −20 °C) and 400 μL of demineralized water (Milli Q). Afterwards, the mixtures were vortexed thoroughly and centrifuged for 15 min at 2,200 rcf at 4 °C. Finally, liquids in equal amount of 200 μL from the aqueous and chloroform layers were transferred into a glass vial for vacuum-dry at room temperature. In addition, Blank samples were also prepared by extraction solution and pooled samples using mixing aliquots from each biological of all the three group taproot samples. Such blank samples were analyzed in each analytic run along with the true samples.

The dried samples were dissolved and derivatized using a two-step procedure involving oximation and silyaltion before injection of GC-MS analysis. First, 30 μL of methoximation mixture consisting of methoxylamine hydrochloride dissolved in pyridine (20 mg∙mL^−1^) were added to the vial, vortexed for 30 s and reacted for 90 min at 37 °C. This was followed by trimethylsilylation with 30 μL of N,O-bis (trimethylsilyl) trifluoroacetamide (BSTFA) containing 1% trimethylchlorosilane (TMCS), keeping the temperature constant at 70 °C for 60 min. Finally, derived samples were cooled to room temperature before injection.

### Gas chromatography-mass spectrometry (GC-MS) analysis

Analysis was performed on an 7890A-5975C GC-MS system (Agilent Technologies, Santa Clara, CA, USA) equipped with an HP-5MS capillary column (30 m × 0.25 mm × 0.25 μm) (Agilent J &W Scientific, Folsom, CA). All the samples and replicates were continuously injected as one batch in random order to discriminate technical from biological variations. Additionally, the prepared pooled samples were used as quality controls (QCs), which were injected at regular intervals (every ten samples) throughout the analytical run to provide a set of data from which the repeatability can be assessed.

Each 1 μL aliquot of the derivatized sample solution was injected in splitless into the GC column by a TriPlus autosampler (Thermo Fisher Scientific), at a constant flow rate of helium of 1 mL·min^−1^. The temperature of the injector was operated isothermally at 280 °C. The mass spectrometry (MS) transfer line temperature to the quadruple was set to 150 °C and the electron impact (EI) ion source ion temperature was 230 °C. Compound elution settings were 2min at 80 °C isothermal, followed by a 10 °C∙min^−1^ oven temperature gradient to a final 325 °C, and then hold for 6 min at 325 °C. The system is then temperature equilibrated for 1 min at 80 °C before injecting the next sample. Ions were generated by a 70 eV electron beam. The spectra were recorded with a scanning range of 50–550 m ∙ z^−1^.

### Data analysis and metabolic pathway construction

The ‘.CDF’ formats files obtained from the mass spectra, were imported into the XCMS software which is based on the R software platform (http://cran.r-project.org). XCMS could be employed to be preprocessed in an automatic way including raw signal extracting, data baseline filtering, peak identification, and integration^55^,^56^. Basically, after alignment with statistic compare component, the ‘.CSV’ file was obtained with three dimension data sets including sample information, retention time and peak intensities. The internal standard was used for data quality control (reproducibility). Internal standards and any known pseudo positive peaks, such as peaks caused by noise, column bleed and BSTFA derivatization procedure, were removed from the data set. Furthermore, the data set was normalized using the sum intensity of the peaks in each sample, which were separately imported into SIMCA-P software package (version 11.0, http://www.umetrics.com/simca). Principal component analysis (PCA) and partial least-squares-discriminant analysis (PLS-DA) were applied to the data after mean-centering and unit variance scaling (UV-scaling). These analyses employed a default seven-fold internal cross validation from which the R^2^ and Q^2^ (goodness of prediction) values, representing the total explained variance and the model predictability, respectively, were extracted^57^,^58^.

Metabolites were identified by comparing the mass-to-charge ratios and the abundance of each compound detected against a standard mass chromatogram in the National Institute of Standards and Technology (NIST) database and the Wiley registry of mass spectral library. Peaks with similarity index more than 70% were tentatively identified as metabolites, whereas those having less than 70% similarity index were considered as unknown metabolites. Two-sample t-test statistics was used for the comparison of the metabolite levels to determine their significant differences. Relationships between the significantly altered metabolites were analyzed with R software (KEGGSOAP package). Metabolites among five steps of reactions were considered related ones, and the visualized networks of metabolite-to-metabolite and gene-to-metabolite were created with cytoscape software^59^,^60^.

## Additional Information

**How to cite this article**: Wang, Y. *et al.* Metabolomic analysis with GC-MS to reveal potential metabolites and biological pathways involved in Pb & Cd stress response of radish roots. *Sci. Rep.* **5**, 18296; doi: 10.1038/srep18296 (2015).

## Supplementary Material

Supplementary Information

Supplementary Dataset 1

Supplementary Dataset 2

Supplementary Dataset 3

Supplementary Dataset 4

Supplementary Dataset 5

Supplementary Dataset 6

Supplementary Dataset 7

Supplementary Dataset 8

## Figures and Tables

**Table 1:** Explanation and predictability values of the principal component analysis (PCA) and partial least squares-discriminate analysis (PLS-DA).

		Control-Pb-Cd	Control-Pb	Control-Cd	Pb-Cd
PCA	R^2^X	0.748	0.732	0.714	0.694
	Q^2^	0.519	0.329	0.412	0.433
PLS-DA	R^2^X	0.560	0.433	0.713	0.671
	R^2^Y	0.516	0.900	0.990	0.476
	Q^2^	0.343	0.650	0.860	0.092

Control-Pb-Cd, analysed using the Pb- and Cd-treated samples as well as the controls;

Control-Pb, analysed using the control and Pb-treated samples;

Control-Cd, analysed using the control and Cd-treated samples;

Pb-Cd, analysed using the Pb- and Cd-treated samples.

**Table 2:** Significant changes in the concentrations of metabolites upon Pb stress exposure in radish roots.

Metabolite		VIP	Fold (Pb-treated/Control)^a^
Sugars	Fructose	1.32	0.61*
	Galactose	1.65	0.59**
	Glucose	1.22	0.24*
	Maltose	1.26	−0.40*
	Inositol	1.88	−0.52**
	Turanose	1.69	−0.80**
	α-D-Glucopyranoside	1.60	−1.29**
	β-D-Glucopyranose	1.75	−1.34**
Amino acids	Isoleucine	1.29	−0.66*
	Serine	1.34	−0.77*
	Glycine	1.16	−0.86*
	Alanine	1.20	−1.80*
Organic acids	Citrate	1.48	2.52*
	Gluconate	1.78	2.16**
	Hexadecanoic acid	1.32	0.68*
	Linoleic acid	1.35	0.53*
	α-Linolenic acid	1.37	0.39*
	Octadecanoic acid	1.22	0.36*
	Oxalic acid	1.68	−0.40**
	Malate	1.32	−0.61*
	Acetimidic acid	1.20	−0.66*
	Decanedioic acid	1.67	−0.95**
Inorganic acids	Phosphoric acid	1.39	−0.77*
	Steride	1.74	0.97**
	Glycerol	1.46	−0.30*
Others	Hydroxylamine	1.48	−0.63*
	Unknown m/z = 73,340,394	1.27	−1.08*
	Sitosterol	1.63	−2.48**

^a^The fold changes in the concentrations of each metabolite between the two groups were calculated using the formula log_2_(Pb-treated/Control). “*” and “**” indicate the difference is significant (*P* < 0.05) and highly significant (*P* < 0.01) compared to the control, respectively. VIP, variable importance in the projection.

**Table 3:** Significant changes in the concentrations of metabolites upon Cd stress exposure in radish roots.

	Metabolite	VIP	Fold (Cd-treated/Control)^a^
Sugars	α-D-Glucopyranoside	1.28	−0.45*
	Turanose	1.43	−0.68*
	Glucose	1.15	−0.68*
	Galactose	1.19	−0.80*
	Inositol	1.29	−0.90*
	Maltose	1.67	−0.91**
	β-D-Glucopyranose	1.34	−1.09*
	Fructose	1.53	−1.26**
Amino acids	Pyroglutamate	1.12	−1.44*
	Glycine	1.32	−1.46*
	Threonine	1.10	−2.10*
	Proline	1.35	−2.26*
	Alanine	1.27	−2.27*
Organic acids	Gluconate	1.11	1.26*
	Octadecanoic acid	1.23	−0.91*
	Hexadecanoic acid	1.31	−0.96*
	Oxalic acid	1.22	−1.03*
	4-Aminobutyrate	1.18	−1.22*
	Citrate	1.12	−1.55*
	Acetimidic acid	1.11	−1.79*
	Nicotinic acid	1.13	−2.14*
	Decanedioic acid	2.02	−2.75**
Inorganic acids	Phosphoric acid	1.62	−1.48**
	Pyrophosphate	1.36	−4.35**
Others	Monostearin	1.37	−0.77**
	Unknown m/z = 154, 174, 243, 73	1.23	−0.84*
	Glycerol	1.61	−0.97**
	Sitosterol	1.20	−1.04*
	Unknown m/z = 183, 198, 73	1.23	−1.29*
	Hydroxylamine	1.69	−1.58**
	Cholesterol	1.68	−1.60**
	Steride	1.64	−2.04**
	Unknown m/z = 340, 394	1.83	−6.00**

^a^The fold changes in the concentrations of each metabolite between the two groups were calculated using the formula log_2_(Cd-treated/Control). “*” and “**” indicate the difference is significant (*P* < 0.05) and highly significant (*P* < 0.01) compared to the control, respectively. VIP, variable importance in the projection.

**Table 4:** KEGG pathway enrichment analysis of the altered metabolites upon Pb and Cd stresses exposure in radish roots.

	Pathway	Adjusted *P*-value	Count
Pb-treated stress	Galactose metabolism	1.86E-04	5
	Starch and sucrose metabolism	0.0036022	4
	Aminoacyl-tRNA biosynthesis	0.0128757	4
	Glyoxylate and dicarboxylate metabolism	0.0236825	3
	Biosynthesis of alkaloids derived from terpenoid and polyketide	0.0253619	3
	Biosynthesis of unsaturated fatty acids	0.0304316	3
	Citrate cycle (TCA cycle)	0.0413384	2
	Biosynthesis of alkaloids derived from ornithine, lysine and nicotinic acid	0.0433843	3
	Carbon fixation in photosynthetic organisms	0.043542	2
Cd-treated stress	Galactose metabolism	2.96E-04	5
	Starch and sucrose metabolism	0.0076641	4
	Aminoacyl-tRNA biosynthesis	0.0180474	4
	Oxidative phosphorylation	0.0497919	2

## References

[b1] Gupta N., Khan D. & Santra S. Heavy metal accumulation in vegetables grown in a long-term wastewater-irrigated agricultural land of tropical India. Environ. Monit. Assess. 184, 6673–6682 (2012). 22131014 10.1007/s10661-011-2450-7

[b2] Liu X. *et al.* Human health risk assessment of heavy metals in soil-vegetable system: a multi-medium analysis. Sci. Total Environ. 463, 530–540 (2013). 23831799 10.1016/j.scitotenv.2013.06.064

[b3] Pourrut B., Shahid M., Douay F., Dumat C. & Pinelli E. Molecular mechanisms involved in lead uptake, toxicity and detoxification in higher plants. In Heavy Metal Stress in Plants (eds Dharmendra K. G., Francisco J. C. & José M. P.) 121–147 (Springer, 2013).

[b4] Seregin I. & Ivanov V. Physiological aspects of cadmium and lead toxic effects on higher plants. Russ. J. Plant Physiol. 48, 523–544 (2001).

[b5] Benavides M. P., Gallego S. M. & Tomaro M. L. Cadmium toxicity in plants. Braz. J. Plant Physiol. 17, 21–34 (2005).

[b6] Sharma P. & Dubey R. S. Lead toxicity in plants. Braz. J. Plant Physiol. 17, 35–52 (2005).

[b7] DalCorso G., Farinati S. & Furini A. Regulatory networks of cadmium stress in plants. Plant Signal Behav. 5, 663–667 (2010). 20404494 10.4161/psb.5.6.11425 PMC3001555

[b8] Thapa G., Sadhukhan A., Panda S. K. & Sahoo L. Molecular mechanistic model of plant heavy metal tolerance. Biometals 25, 489–505 (2012). 22481367 10.1007/s10534-012-9541-y

[b9] Gupta O., Sharma P., Gupta R. & Sharma I. MicroRNA mediated regulation of metal toxicity in plants: present status and future perspectives. Plant Mol. Biol. 84, 1–18 (2014). 23975146 10.1007/s11103-013-0120-6

[b10] Fell D. A. Beyond genomics. Trends Genet. 17, 680–682 (2001). 11718905 10.1016/s0168-9525(01)02521-5

[b11] Urano K., Kurihara Y., Seki M. & Shinozaki K. ‘Omics’ analyses of regulatory networks in plant abiotic stress responses. Curr. Opin. Plant Biol. 13, 132–138 (2010). 20080055 10.1016/j.pbi.2009.12.006

[b12] Booth S. C., Workentine M. L., Weljie A. M. & Turner R. J. Metabolomics and its application to studying metal toxicity. Metallomics 3, 1142–1152 (2011). 21922109 10.1039/c1mt00070e

[b13] Patti G. J., Yanes O. & Siuzdak G. Innovation: Metabolomics: the apogee of the omics trilogy. Nat. Rev. Mol. Cell Biol. 13, 263–269 (2012). 22436749 10.1038/nrm3314 PMC3682684

[b14] Chagoyen M. & Pazos F. Tools for the functional interpretation of metabolomic experiments. Brief. Bioinform. 14, 737–744 (2012). 23063930 10.1093/bib/bbs055

[b15] Dunn W. B. & Ellis D. I. Metabolomics: current analytical platforms and methodologies. TrAC-Trends Analyt. Chem. 24, 285–294 (2005).

[b16] Saito K. & Matsuda F. Metabolomics for functional genomics, systems biology, and biotechnology. Annu. Rev. Plant Biol. 61, 463–489 (2010). 19152489 10.1146/annurev.arplant.043008.092035

[b17] Nikiforova V. J. *et al.* Systems rebalancing of metabolism in response to sulfur deprivation, as revealed by metabolome analysis of *Arabidopsis* plants. Plant Physiol. 138, 304–318 (2005). 15834012 10.1104/pp.104.053793 PMC1104185

[b18] Le Lay P. *et al.* Metabolomic, proteomic and biophysical analyses of *Arabidopsis thaliana* cells exposed to a caesium stress. Influence of potassium supply. Biochimie 88, 1533–1547 (2006). 16716483 10.1016/j.biochi.2006.03.013

[b19] Bailey N. J., Oven M., Holmes E., Nicholson J. K. & Zenk M. H. Metabolomic analysis of the consequences of cadmium exposure in *Silene cucubalus* cell cultures via ^1^H NMR spectroscopy and chemometrics. Phytochemistry 62, 851–858 (2003). 12590112 10.1016/s0031-9422(02)00719-7

[b20] Hédiji H. *et al.* Effects of long-term cadmium exposure on growth and metabolomic profile of tomato plants. Ecotoxicol. Environ. Saf. 73, 1965–1974 (2010). 20846723 10.1016/j.ecoenv.2010.08.014

[b21] Kieffer P. *et al.* Combining proteomics and metabolite analyses to unravel cadmium stress-response in poplar leaves. J. Proteome Res. 8, 400–417 (2008). 19072159 10.1021/pr800561r

[b22] Uraguchi S. *et al.* Root-to-shoot Cd translocation via the xylem is the major process determining shoot and grain cadmium accumulation in rice. J. Exp. Bot. 60, 2677–2688 (2009). 19401409 10.1093/jxb/erp119 PMC2692013

[b23] Kapourchal S. A., Kapourchal S. A., Pazira E. & Homaee M. Assessing radish (*Raphanus sativus* L.) potential for phytoremediation of lead-polluted soils resulting from air pollution. Plant Soil Environ. 55, 202–206 (2009).

[b24] Wang Y. *et al.* Transcriptome profiling of radish (*Raphanus sativus* L.) root and identification of genes involved in response to lead (Pb) stress with next generation sequencing. PloS One 8, e66539 (2013). 23840502 10.1371/journal.pone.0066539 PMC3688795

[b25] Xu L. *et al.* Genome-wide identification and characterization of cadmium-responsive microRNAs and their target genes in radish (*Raphanus sativus* L.) roots. J. Exp. Bot. 64, 4271–4287 (2013). 24014874 10.1093/jxb/ert240 PMC3808317

[b26] Schauer N. & Fernie A. R. Plant metabolomics: towards biological function and mechanism. Trends Plant Sci. 11, 508–516 (2006). 16949327 10.1016/j.tplants.2006.08.007

[b27] Woo H. M. *et al.* Mass spectrometry based metabolomic approaches in urinary biomarker study of women’s cancers. Clin. Chim. Acta 400, 63–69 (2009). 19010317 10.1016/j.cca.2008.10.014

[b28] El-Beltagi H. & Mohamed A. Changes in non protein thiols, some antioxidant enzymes activity and ultrastructural alteration in radish plant (*Raphanus sativus* L.) grown under lead toxicity. Not. Bot. Horti Agrobot. Cluj Napoca 38, 76–85 (2010).

[b29] Inoue H. *et al.* Properties of lead deposits in cell walls of radish (*Raphanus sativus*) roots. J. Plant Res. 126, 51–61 (2013). 22644314 10.1007/s10265-012-0494-6

[b30] He L. L. *et al.* Physiological responses of radish (*Raphanus Sativus* L.) to lead stress. In *Bioinformatics and Biomedical Engineering*, 2008. ICBBE 2008. The 2nd International Conference on 4602-4605 (IEEE, 2008).

[b31] Wang Y. *et al.* Transport, ultrastructural localization, and distribution of chemical forms of lead in radish (*Raphanus sativus* L.). Front. Plant Sci. 6, 293 (2015). 26005445 10.3389/fpls.2015.00293 PMC4424845

[b32] Xu L. *et al.* *De novo* sequencing of root transcriptome reveals complex cadmium-responsive regulatory networks in radish (*Raphanus sativus* L.). Plant Sci. 236, 313–323 (2015). 26025544 10.1016/j.plantsci.2015.04.015

[b33] Yang J. *et al.* Cadmium uptake character and stress effect on the growth and antioxidant enzymes activities in *Raphanus sativus*. Acta Hortic. 767, 249 (2008).

[b34] Arbona V., Manzi M., Ollas C. d. & Gómez-Cadenas A. Metabolomics as a tool to investigate abiotic stress tolerance in plants. Int. J. Mol. Sci. 14, 4885–4911 (2013). 23455464 10.3390/ijms14034885 PMC3634444

[b35] Ariza J. G., García-Barrera T., García-Sevillano M., González-Fernández M. & Gómez-Jacinto V. Metallomics and metabolomics of plants under environmental stress caused by metals. In Heavy Metal Stress in Plants (eds Dharmendra K. G., Francisco J. C. & José M. P.) 173–201 (Springer, 2013).

[b36] Brunetti C., George R. M., Tattini M., Field K. & Davey M. P. Metabolomics in plant environmental physiology. J. Exp. Bot. 64, 4011–4020 (2013). 23922358 10.1093/jxb/ert244

[b37] Tanyolac D., Ekmekçi Y. & Ünalan Ş. Changes in photochemical and antioxidant enzyme activities in maize (*Zea mays* L.) leaves exposed to excess copper. Chemosphere 67, 89–98 (2007). 17109927 10.1016/j.chemosphere.2006.09.052

[b38] Aziz T. *et al.* A mini review on lead (Pb) toxicity in plants. J. Biol. life Sci. 6, 91–101 (2015).

[b39] Hall J. Cellular mechanisms for heavy metal detoxification and tolerance. J. Exp. Bot. 53, 1–11 (2002). 11741035

[b40] Jones D., Dennis P., Owen A. & Van Hees P. Organic acid behavior in soils-misconceptions and knowledge gaps. Plant Soil 248, 31–41 (2003).

[b41] Kochian L., Pineros M. & Hoekenga O. The physiology, genetics and molecular biology of plant aluminum resistance and toxicity. Plant Soil 274, 175–195(2005).

[b42] Ma J. F., Chen Z. C. & Shen R. F. Molecular mechanisms of Al tolerance in gramineous plants. Plant Soil 381, 1–12 (2014).

[b43] Ma J. F., Ryan P. R. & Delhaize E. Aluminium tolerance in plants and the complexing role of organic acids. Trends Plant Sci. 6, 273–278 (2001). 11378470 10.1016/s1360-1385(01)01961-6

[b44] Kavita B., Shukla S., Kumar G. N. & Archana G. Amelioration of phytotoxic effects of Cd on mung bean seedlings by gluconic acid secreting rhizobacterium *Enterobacter asburiae* PSI3 and implication of role of organic acid. World J. Microbiol. Biotechnol. 24, 2965–2972 (2008).

[b45] Ghnaya T. *et al.* Implication of organic acids in the long-distance transport and the accumulation of lead in *Sesuvium portulacastrum* and *Brassica juncea*. Chemosphere 90, 1449–1454 (2013). 23026160 10.1016/j.chemosphere.2012.08.061

[b46] Rai V. Role of amino acids in plant responses to stresses. Biol. Plant. 45, 481–487 (2002).

[b47] Hirai M. Y. *et al.* Elucidation of gene-to-gene and metabolite-to-gene networks in *Arabidopsis* by integration of metabolomics and transcriptomics. J. Biol. Chem. 280, 25590–25595 (2005). 15866872 10.1074/jbc.M502332200

[b48] Estrella-Gómez N. E., Sauri-Duch E., Zapata-Pérez O. & Santamaría J. M. Glutathione plays a role in protecting leaves of *Salvinia minima* from Pb^2+^ damage associated with changes in the expression of SmGS genes and increased activity of GS. Environ. Exp. Bot. 75, 188–194 (2012).

[b49] Jozefczak M., Remans T., Vangronsveld J. & Cuypers A. Glutathione is a key player in metal-induced oxidative stress defenses. Int. J. Mol. Sci. 13, 3145–3175 (2012). 22489146 10.3390/ijms13033145 PMC3317707

[b50] Sappl P. G. *et al.* The *Arabidopsis* glutathione transferase gene family displays complex stress regulation and co-silencing multiple genes results in altered metabolic sensitivity to oxidative stress. Plant J. 58, 53–68 (2009). 19067976 10.1111/j.1365-313X.2008.03761.x

[b51] Gupta D., Huang H., Yang X., Razafindrabe B. & Inouhe M. The detoxification of lead in *Sedum alfredii* H. is not related to phytochelatins but the glutathione. J. Hazard. Mater. 177, 437–444 (2010). 20047791 10.1016/j.jhazmat.2009.12.052

[b52] Xu L. *et al.* Genetic linkage map construction and QTL mapping of cadmium accumulation in radish (*Raphanus sativus* L.). Theor. Appl. Genet. 125, 659–670 (2012). 22491896 10.1007/s00122-012-1858-y

[b53] Lisec J., Schauer N., Kopka J., Willmitzer L. & Fernie A. R. Gas chromatography mass spectrometry-based metabolite profiling in plants. Nat. Protoc. 1, 387 (2006). 17406261 10.1038/nprot.2006.59

[b54] Wu D. *et al.* Tissue metabolic responses to salt stress in wild and cultivated barley. PloS One 8, e55431 (2013). 23383190 10.1371/journal.pone.0055431 PMC3561194

[b55] Smith C. A., Want E. J., O’Maille G., Abagyan R. & Siuzdak G. XCMS: processing mass spectrometry data for metabolite profiling using nonlinear peak alignment, matching, and identification. Anal. Chem. 78, 779–787 (2006). 16448051 10.1021/ac051437y

[b56] Tautenhahn R., Patti G. J., Rinehart D. & Siuzdak G. XCMS Online: a web-based platform to process untargeted metabolomic data. Anal. Chem. 84, 5035–5039 (2012). 22533540 10.1021/ac300698c PMC3703953

[b57] Eriksson L., Andersson P. L., Johansson E. & Tysklind M. Megavariate analysis of environmental QSAR data. Part I-A basic framework founded on principal component analysis (PCA), partial least squares (PLS), and statistical molecular design (SMD). Mol. Divers. 10, 169–186 (2006). 16770514 10.1007/s11030-006-9024-6

[b58] Zhang J., Zhang Y., Du Y., Chen S. & Tang H. Dynamic metabonomic responses of tobacco (*Nicotiana tabacum*) plants to salt stress. J. Proteome Res. 10, 1904–1914 (2011). 21323351 10.1021/pr101140n

[b59] Shannon P. *et al.* Cytoscape: a software environment for integrated models of biomolecular interaction networks. Genome Res. 13, 2498–2504 (2003). 14597658 10.1101/gr.1239303 PMC403769

[b60] Lopes C. T. *et al.* Cytoscape Web: an interactive web-based network browser. Bioinformatics 26, 2347–2348 (2010). 20656902 10.1093/bioinformatics/btq430 PMC2935447

