# Supplementary material for: Metabolomic analysis with GC-MS to reveal potential metabolites and biological pathways involved in Pb & Cd stress response of radish roots
Source: Sci Rep. 2015 Dec 17;5:18296. doi: 10.1038/srep18296 (PMC4682141; doi:10.1038/srep18296)
Supplement: Supplementary Information [file srep18296-s1.pdf]

Supplementary information

Metabolomic analysis with GC-MS to reveal potential metabolites and biological pathways involved in Pb & Cd stress response of radish roots

Yan Wang, Liang Xu, Hong Shen, Juanjuan Wang, Wei Liu, Xianwen Zhu, Ronghua Wang, Xiaochuan Sun, Liwang Liu*

Supplementary Figures

Supplementary Fig. S1: GC-MS spectra for a typical sample in control (A), Pb-treated (B) and Cd- treated (C) roots.

Supplementary Fig. S2: Metabolite-to-metabolite networks upon Pb (A) or Cd (B) stress exposure in radish roots.

Supplementary Fig. S3: Primary metabolic and transcriptional changes during Pb exposure in radish roots. Metabolites in red and blue indicate significant ($P<0.05$) up- and down-accumulation in Pb-treated samples, respectively. Gene products were shown in square frame. The solid lines and dotted lines represent direct and indirect interactions, respectively.

Supplementary Fig. S4: Primary metabolic and transcriptional changes during Cd exposure in radish roots. Metabolites in red and blue indicate significant ($P<0.05$) up- and down-accumulation in Cd-treated samples, respectively. Gene products were shown in square frame. The solid lines and dotted lines represent direct and indirect interactions, respectively.

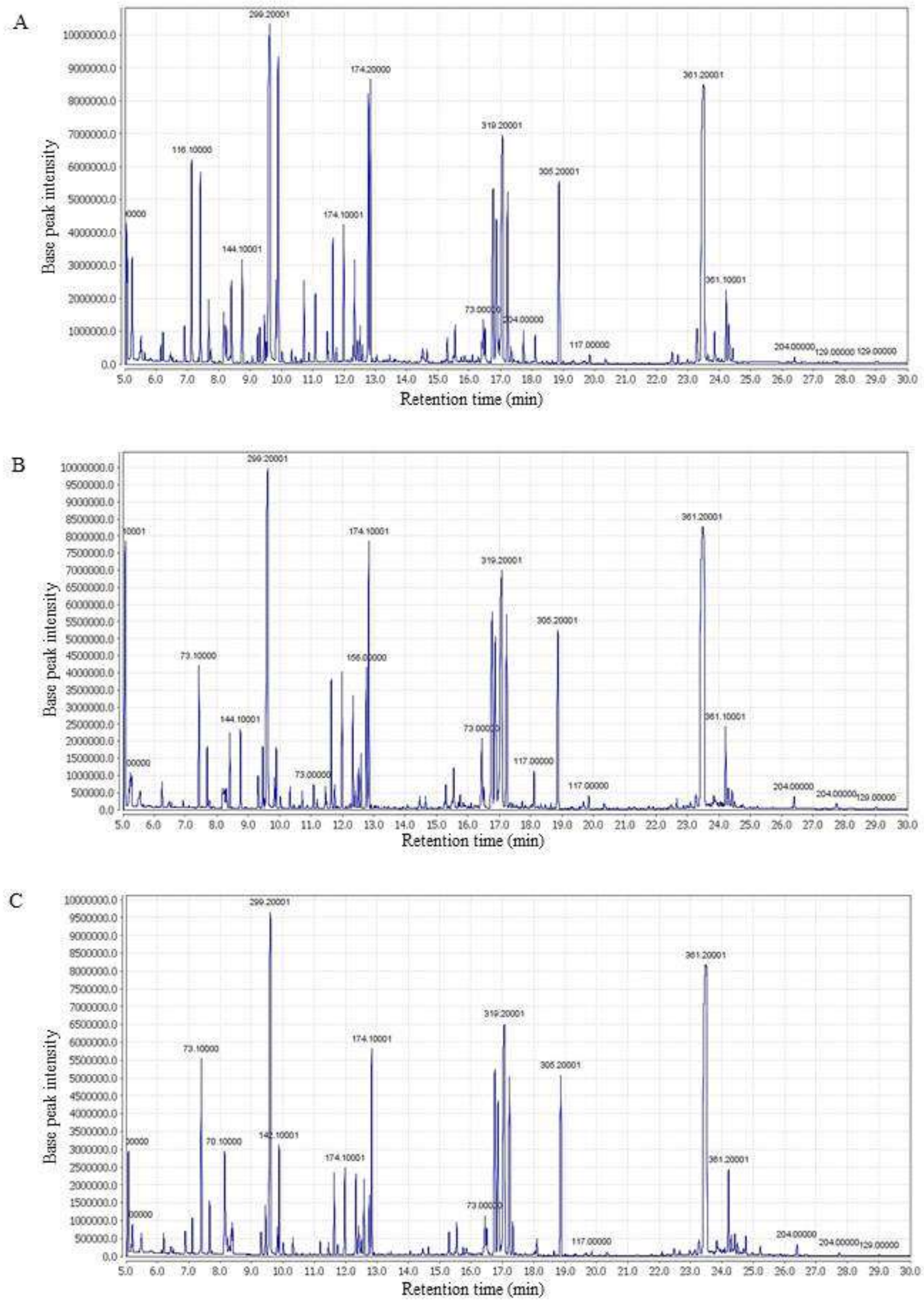

Supplementary Fig. S1 GC-MS spectra for a typical sample in control (A), Pb-treated (B) and Cd- treated (C) roots.

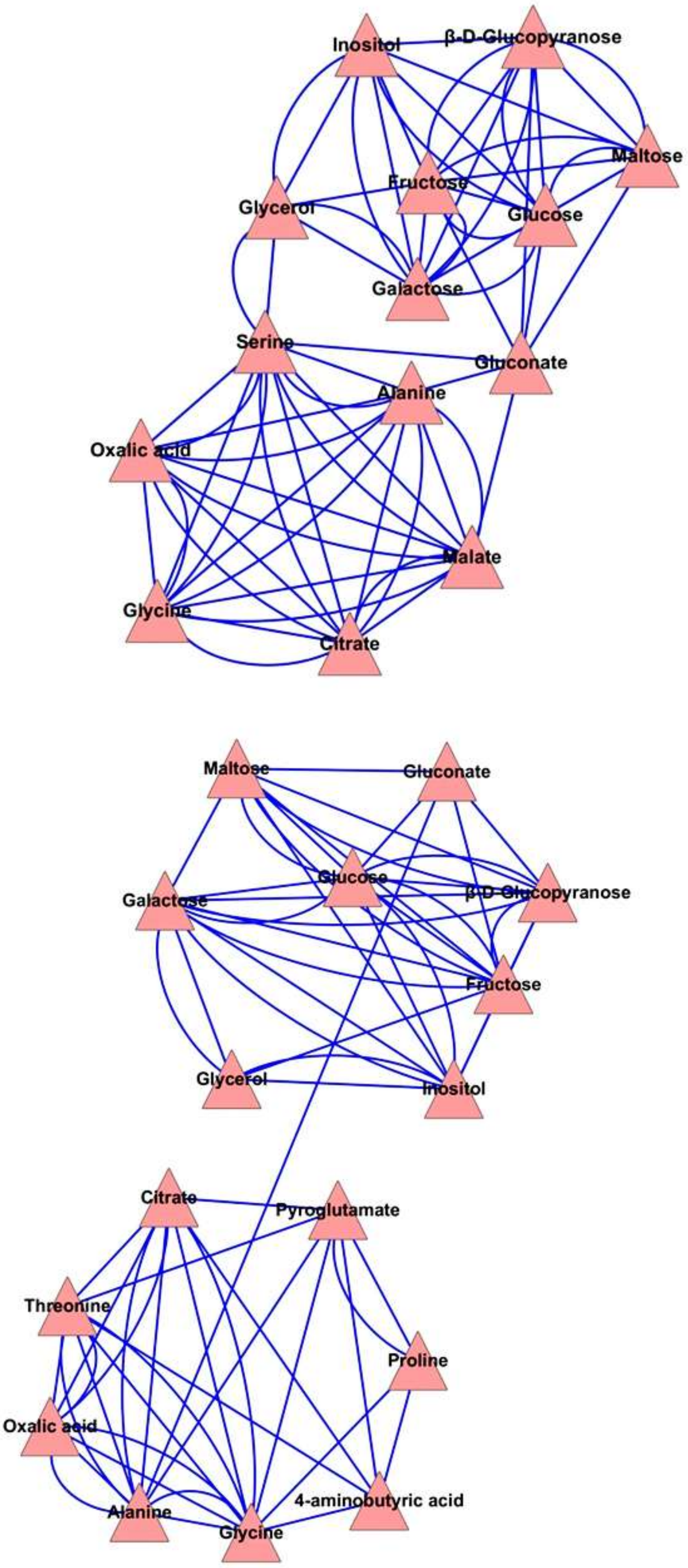

Supplementary Fig. S2 Metabolite-to-metabolite networks upon Pb (A) or Cd (B) stress exposure in radish roots.

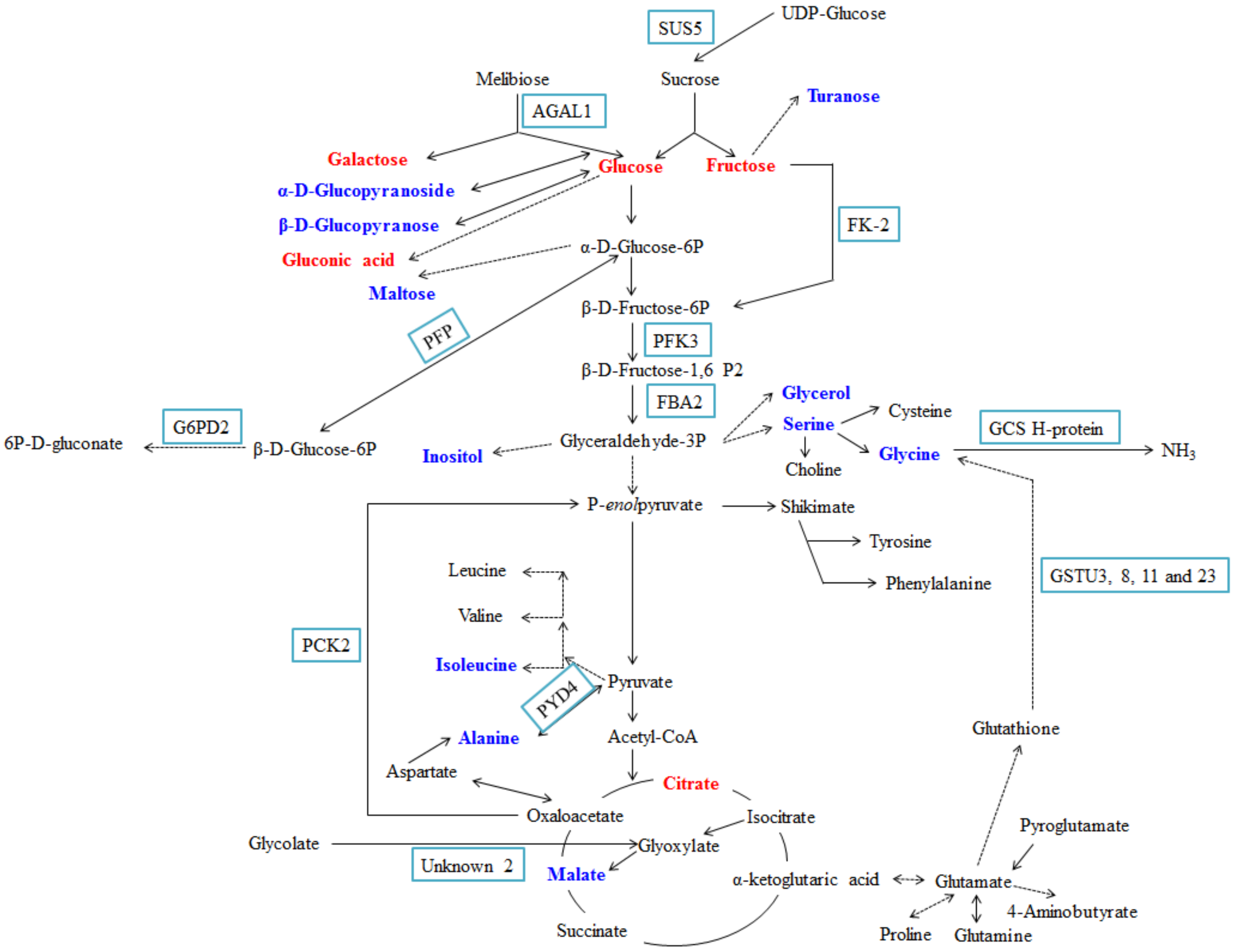

UDP-Glucose
SUS5
Sucrose
Melibiose
AGAL1
Turanose
Galactose
Glucose
Fructose
α-D-Glucopyranoside
β-D-Glucopyranose
Gluconic acid
Maltose
FK-2
α-D-Glucose-6P
β-D-Fructose-6P
PFP
PFK3
β-D-Fructose-1,6 P2
FBA2
Glycerol
Cysteine
Serine
GCS H-protein
Glycine
NH3
Choline
G6PD2
6P-D-gluconate
β-D-Glucose-6P
Inositol
Glyceraldehyde-3P
P-enolpyruvate
Shikimate
Tyrosine
Phenylalanine
GSTU3, 8, 11 and 23
Leucine
Valine
PCK2
Isoleucine
PYD4
Pyruvate
Alanine
Acetyl-CoA
Glutathione
Aspartate
Citrate
Oxaloacetate
Isocitrate
Glycolate
Glyoxylate
Pyroglutamate
Unknown 2
Malate
α-ketoglutaric acid
Glutamate
4-Aminobutyrate
Succinate
Proline
Glutamine

Supplementary Fig. S3 Primary metabolic and transcriptional changes during Pb exposure in radish roots. Metabolites in red and blue indicate significant (P<0.05) up- and down-accumulation in Pb-treated samples, respectively. Gene products were shown in square frame. The solid lines and dotted lines represent direct and indirect interactions, respectively.

PFP, pyrophosphate-fructose-6-phosphate1-phosphotransferase; GSTU3, 8, 11 and 23, glutat-hione S-transferase TAU 3, 8, 11 and 23; GCS H-protein, glycine cleavage system H protein; unknown2, Aldolase-type TIM barrel family protein; PYD4, pyrimidine 4; AGAL1, alpha-galactosidase 1; PFK3, 6-phosphofructokinase 3; FBA2, mmfructose-bisphosphate aldolase, class I; G6PD2, glucose-6-phosphate dehydrogenase 2; PCK2, phosphoenolpyruvate carboxykinase 2; SUS5, sucrose synthase 5; FK-2, fructokinase.

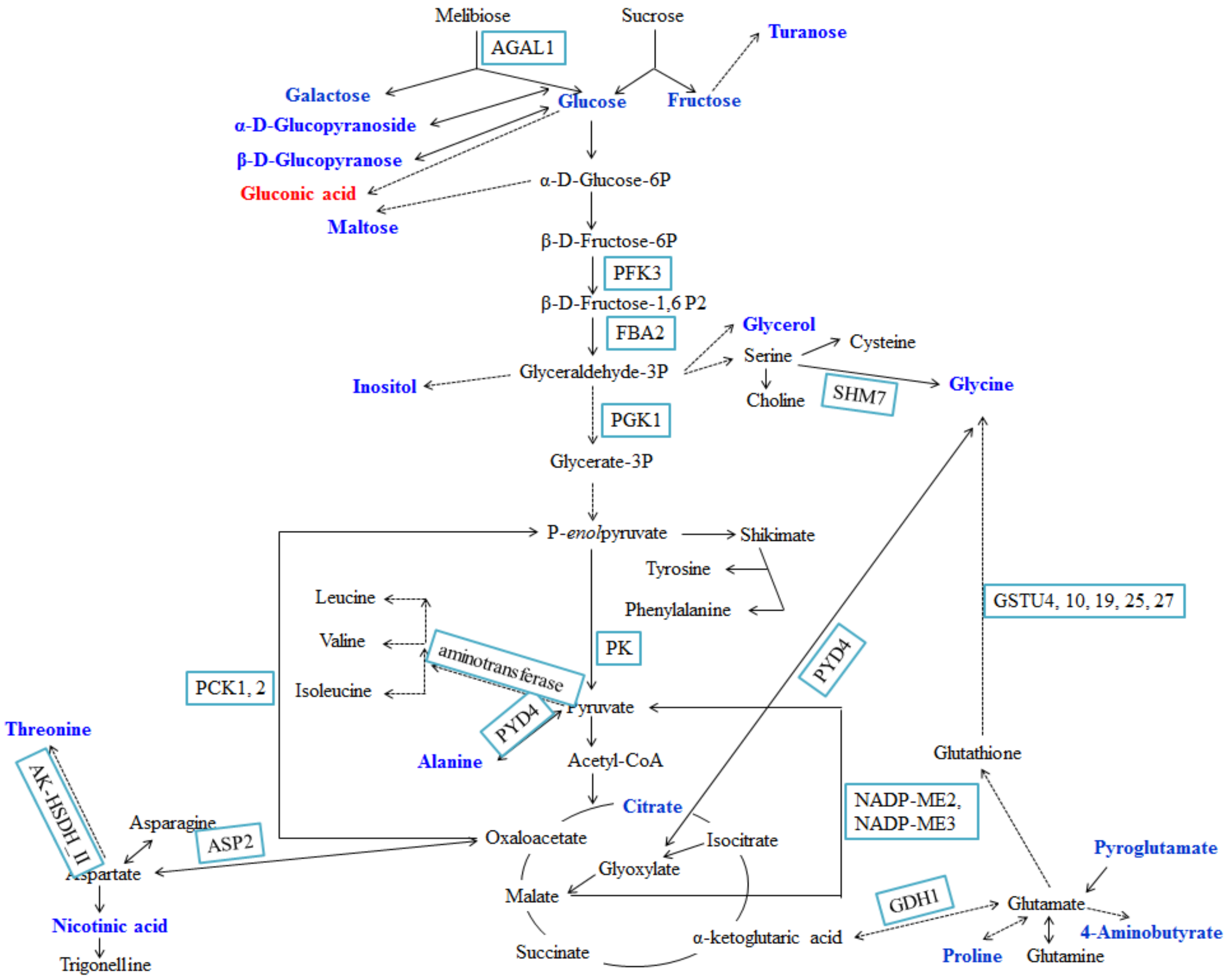
Melibiose
Sucrose
AGAL1
Turanose
Galactose
Glucose
Fructose
α-D-Glucopyranoside
β-D-Glucopyranose
Gluconic acid
α-D-Glucose-6P
Maltose
β-D-Fructose-6P
PFK3
β-D-Fructose-1,6 P2
FBA2
Glycerol
Serine
Cysteine
Inositol
Glyceraldehyde-3P
Choline
SHM7
Glycine
PGK1
Glycerate-3P
P-enolpyruvate
Shikimate
Tyrosine
Phenylalanine
Leucine
Valine
Isoleucine
aminotransferase
PK
PCK1, 2
GSTU4, 10, 19, 25, 27
PYD4
Pyruvate
Threonine
PYD4
Alanine
Acetyl-CoA
Glutathione
AK-HSDH_II
Citrate
NADP-ME2,
NADP-ME3
Asparagine
ASP2
Oxaloacetate
Isocitrate
Aspartate
Glyoxylate
Pyroglutamate
Malate
GDH1
Glutamate
Nicotinic acid
4-Aminobutyrate
Succinate
α-ketoglutaric acid
Proline
Glutamine
Trigonelline

Supplementary Fig. S4 Primary metabolic and transcriptional changes during Cd exposure in radish roots. Metabolites in red and blue indicate significant (P<0.05) up- and down-accumulation in Cd-treated samples, respectively. Gene products were shown in square frame. The solid lines and dotted lines represent direct and indirect interactions, respectively.

AK-HSDH_II, bifunctional aspartokinase/homoserine dehydrogenase 1; ASP2, aspartate aminotr-ansferase; GDH1, glutamate dehydrogenase 1; GSTU4, 10, 19, 25, 27, glutathione S-transferase TAU 4, 10, 19, 25, 27; NADP-ME2,3 malate dehydrogenase (oxaloacetate-decarboxylating ($NADP^+$); PCK1, phosphoenolpyruvate carboxykinase 1; PGK1, phosphoglycerate kinase 1; SHM7, serine hydroxymethyltransferase 7; PK, Pyruvate kinase.

Supplementary Tables

Supplementary Table S1: The identified Pb-responsive metabolites from the GC-MS (See Excel file).

Supplementary Table S2: The identified Cd-responsive metabolites from the GC-MS (See Excel file).

Supplementary Table S3: The KEGG pathways of the altered metabolites exposure to Pb stress in radish roots (See Excel file).

Supplementary Table S4: The KEGG pathways of the altered metabolites exposure to Cd stress in radish roots (See Excel file).

Supplementary Table S5: The overlapped pathways of the differentially expressed genes and significantly altered metabolites exposure to Pb stress in radish roots (See Excel file).

Supplementary Table S6: The changed genes and metabolites involved in gene-to-metabolite network upon Pb stress exposure in radish (See Excel file).

Supplementary Table S7: The overlapped pathways of the differentially expressed genes and significantly altered metabolites exposure to Cd stress in radish roots (See Excel file).

Supplementary Table S8: The changed genes and metabolites involved in gene-to-metabolite network upon Cd stress exposure in radish (See Excel file).
